# Understanding Dynamics of Information Transmission in *Drosophila melanogaster* Using a Statistical Modeling Framework for Longitudinal Network Data (the RSiena Package)

**DOI:** 10.3389/fpsyg.2016.00539

**Published:** 2016-04-20

**Authors:** Cristian Pasquaretta, Elizabeth Klenschi, Jérôme Pansanel, Marine Battesti, Frederic Mery, Cédric Sueur

**Affiliations:** ^1^Département Ecologie, Physiologie et Ethologie, Centre National de la Recherche ScientifiqueStrasbourg, France; ^2^Institut Pluridisciplinaire Hubert Curien, Université de StrasbourgStrasbourg, France; ^3^Evolution, Génomes, Comportement and Ecologie, Centre National de la Recherche Scientifique, Institut de Recherche pour le Développement, Université Paris-Sud, Université Paris-SaclayGif-sur-Yvette, France

**Keywords:** social network analysis, social learning, information transmission, actor-oriented model, social interactions

## Abstract

Social learning – the transmission of behaviors through observation or interaction with conspecifics – can be viewed as a decision-making process driven by interactions among individuals. Animal group structures change over time and interactions among individuals occur in particular orders that may be repeated following specific patterns, change in their nature, or disappear completely. Here we used a stochastic actor-oriented model built using the RSiena package in R to estimate individual behaviors and their changes through time, by analyzing the dynamic of the interaction network of the fruit fly *Drosophila melanogaster* during social learning experiments. In particular, we re-analyzed an experimental dataset where uninformed flies, left free to interact with informed ones, acquired and later used information about oviposition site choice obtained by social interactions. We estimated the degree to which the uninformed flies had successfully acquired the information carried by informed individuals using the proportion of eggs laid by uninformed flies on the medium their conspecifics had been trained to favor. Regardless of the degree of information acquisition measured in uninformed individuals, they always received and started interactions more frequently than informed ones did. However, information was efficiently transmitted (i.e., uninformed flies predominantly laid eggs on the same medium informed ones had learn to prefer) only when the difference in contacts sent between the two fly types was small. Interestingly, we found that the degree of reciprocation, the tendency of individuals to form mutual connections between each other, strongly affected oviposition site choice in uninformed flies. This work highlights the great potential of RSiena and its utility in the studies of interaction networks among non-human animals.

## Introduction

Social learning, defined as the transmission of behaviors through observation or interaction with conspecifics (Heyes, 1994), has been extensively studied in many different taxa (e.g., bumblebees, Leadbeater and Chittka, 2005; rodents, Galef and Clark, 1971; sperm whales, Weilgart and Whitehead, 1997; primates Whiten, 2000; van de Waal et al., 2013). Because of the advantages and drawbacks traditionally associated with social learning, it was first described as a fitted adaptation in environments where it is significantly less costly than individual, trial-and-error learning (Boyd and Richerson, 1988).

Individuals should not look for information indiscriminately within their group, as some individuals may hold a piece of information that is irrelevant, outdated, or misleading to the receiver (Kendal et al., 2005; Enquist et al., 2007; Rieucau and Giraldeau, 2011). Social learning strategies thus rely on the identification of the most successful individuals as best potential sources of information inside the group, taking into account the associated risk of inaccuracy (Kendal et al., 2005). In other words, some individuals may contact, or be contacted by, more members of the group or more often. In this regard, the social structure that emerges from inter-individual interactions is crucial in understanding how information is transmitted and if this transmission is efficient (Pasquaretta et al., 2014).

A social structure can be represented as a network where individuals are nodes connected by edges representing one or several types of interactions or relationships occurring among them (Wasserman and Faust, 1994). As summarized by Newman (2003), social network analysis can be used to draw and visualize networks, run statistical analysis of network properties, model networks, and predict the behavior of individuals or patterns in the networks. Connections among individuals (e.g., interactions) are channels for the transmission of information from one individual to another, and they are continually rearranged over time (Blonder et al., 2012). Each individual behaves differently during such a process, transmitting or receiving information from different conspecifics at different times.

Recently temporal dynamic approaches have been developed to study the structural changes occurring in a network along discrete and/or continuous time scales. Such methods are well suited to study social processes in animals such as communication, disease transmission, social learning, and many others (Pinter-Wollman et al., 2014). In particular, a temporal network approach may help to clarify how the dynamics of animal interactions modify network topology and relates to information flow (Charbonneau et al., 2013) and learning (Skyrms, 2009). Some of these methods come from human social science and have principally been developed to predict behaviors based on social structure (Steglich et al., 2006; Mercken et al., 2010; Snijders et al., 2010b; Schaefer et al., 2011). In this context, the use of the dynamic actor-oriented model developed in the R package RSiena (Ripley et al., 2013a) provides powerful estimations of individual behaviors and their changes through time. These methods, developed in the RSiena package (Ripley et al., 2013a), allow users to perform a wide range of data analysis on the same platform used for dynamic modeling operations. Despite having been developed for human social sciences, such techniques can prove very useful in studying the dynamics of interactions in animal societies as they integrate temporal analysis into an actor-oriented modeling approach (described in Snijders et al., 2010b). These methods assume that the dynamics of network structure are the product of a multitude of small changes happening continuously, of which the results are observed over a discrete time line. Moreover, the evaluation of the dynamic processes occurring inside a social structure is strongly dependent on the timescale used. Blonder and Dornhaus (2011) recently underlined the importance of using an appropriate timescale to observe information flow, and a study on the ant *Temnothorax rugatulus* had also shown a discrepancy in the results obtained depending on the time-scale used (Charbonneau et al., 2013). Indeed at large timescales, it was observed that information flow within the colony was slower than expected, whereas at smaller timescales it was faster, suggesting that the network facilitated local rather than global information transmission.

In this work, we performed a social network analysis using the RSiena package to evaluate the dynamic of social interactions during social learning experiments, using the gregarious species *Drosophila melanogaster*, which has already been demonstrated to rely on social learning regarding oviposition site preferences (Sarin and Dukas, 2009; Battesti et al., 2012). Schneider et al. (2012) have demonstrated the existence of non-random interaction networks in wild-type individuals in this species, and more recently, experiments performed by Battesti et al. (2012) provided evidence for social learning through the observation of oviposition site preference. In their protocol, they used uninformed flies that were left free to interact with individuals that had been trained to favor one of two oviposition media. Their results showed that, after that interaction phase, uninformed flies significantly favored the oviposition site the other individuals had been trained to prefer. In another recently published work we have also showed that uninformed flies, in addition to favoring the oviposition site the other individuals had been trained to, can also clearly avoid the information received by laying their eggs on the opposite site informed flies were trained to choose (Pasquaretta et al., 2016). The “avoid” or “follow” decision appeared to be driven by the homogeneity of contact behaviors among informed flies; that homogeneity was a *condition sine qua non* for the information to be successfully followed.

The current study aims at evaluating individual behaviors that could explain the varying outcome of social transmission by studying the dynamics of interactions among flies. RSiena was used to highlight the impact of social network dynamics on the diffusion of information. While fruit flies use olfactory and gustatory sensory organs to identify the sex of encountered individuals (Fernández and Kravitz, 2013), they seem to strongly rely on direct mechanosensory interactions as well in order to elicit responses from flies (Ramdya et al., 2015). Since the success of social transmission strongly relies on interactions between informed and uninformed flies (Battesti et al., 2012) and is affected by direct contacts among individuals (Battesti et al., 2015; Pasquaretta et al., 2016), we expect to find a discrepancy in the way these two fly types (i.e., informed and uninformed) interacted in accordance with the transmission outcome. Uninformed flies show an increase in their activity level when facing informed individuals in the arena (Battesti et al., 2015), which may directly affect the rate of contacts experienced. Here, we focus on the analysis of the numbers of contacts sent and received (also known as *outdegree* and *indegree* in social network analysis) by both informed and uninformed flies, and we expect to find higher *outdegree* and *indegree* measures in uninformed flies compared to informed ones. We evaluated the presence of homophily – the tendency of individuals to associate with similar conspecifics – in the networks to assess the presence of a possible bias in interaction exchanges within classes. Indeed, significantly high levels of homophily for both classes suggest the existence of closed subgroups where information may get fixed (in the case of homophily in informed flies) or never transmitted (in the case of homophily in uninformed flies). Finally, in order to evaluate the impact of both individual and neighboring degrees on the probability of receiving and starting future interactions, we estimated the effect of being linked to individuals that have received many contacts and the effect of being linked to individuals that have sent many contacts in the transmission arena.

## Materials and Methods

### Behavioral Experiments

Using already published data on information transmission in flies (Battesti et al., 2015), we processed recordings of the social transmission phases of the experiments to identify interactions between individuals and analyze the resulting social networks. In those experiments eight female drosophilae were conditioned by introducing them into a 120 mm × 50 mm × 90 mm plastic cage and leaving them for 8 h with the choice between two oviposition media (3 ml contained in 30 mm diameter Petri dishes with 20 g/l of sucrose, 10 g/l of agar and 6 ml/l of artificial banana or strawberry flavors, la Gazignaire SA). Females were trained to prefer one oviposition site over the other with the help of quinine, an alkaloid known to induce gustatory repulsion in fruit flies (Quinn et al., 1974); 50% of the replicates had quinine in the banana-flavored medium and 50% had quinine in the strawberry-flavored medium. Following this conditioning phase, the eight informed females were introduced together with four uninformed individuals in a semi-opaque white polyoxymethylene (Delrin) arena (diameter 100 mm; height 3 mm) covered with transparent Plexiglas (design based on previous work by Simon and Dickinson, 2010). After a social transmission phase lasting 4 h, flies were gently removed from the arena and immediately introduced into a plastic cage containing two oviposition sites again, this time using quinine-free banana- and strawberry-flavored media. We subsequently calculated the proportion of eggs laid by uninformed individuals on each medium at the end of each experiment. Two conditions were then defined: (1) “Followed” (flies followed the information gathered by informed individuals) when uninformed flies mostly laid their eggs on the medium informed flies had learn to prefer in the conditioning phase (proportion of eggs laid on the informed medium by uninformed flies greater than 0.8, *N* = 29, of which 16 on strawberry-flavored medium), and (2) “Avoided” (flies avoided the information gathered by informed individuals) when they laid their eggs in majority on the other medium (proportion on informed medium lower than 0.2, *N* = 19, of which 8 on strawberry-flavored medium).

### Video Analysis

The social transmission phases were recorded using a camera placed vertically above the arena. Using the Ctrax software (Branson et al., 2009), the movements of each individual were automatically followed and its coordinates in the arena recorded for each frame of the video, at a rate of 10 frames per second. Using these coordinates as our raw data, we constructed interaction matrices for each experiment using an automated code we specifically developed in R (code available under request). To this end, we defined an interaction between two individuals based on spatial and temporal constraints: proximity between two flies had to (1) be smaller than 1.1 average body lengths and (2) last for more than five frames of the video (i.e., 0.5 s). These thresholds were derived from several preliminary assumptions and observations. We calculated the average body length of the individuals for each video based on the body length measured by Ctrax for each individual in each frame. Flies can interact using different angles of approach, but the largest distance between two flies would only occur in the case of an approach from the front or rear (for a better graphical explanation of the interaction see Figure 2 in Pasquaretta et al., 2016). In these types of interactions, the distance between the centers of the two individuals will thus be equal to one body length in the case of direct head-to-head contact. We added a 10 percent margin to account for possible contacts between antennae or front legs (structures which are too small for Ctrax to be detected) even when bodies were not in direct contact. Secondly, our temporal criteria to define interactions were based on our observations that proximity lasting under 0.5 s usually corresponded to individuals crossing paths without stopping to interact. Moreover, to discriminate between the initiator and the receiver, we estimated the mean speed of the individuals during an interval lasting four time-frames and preceding each contact by calculating their traveled distance during this interval; the initiator was defined as the fastest individual between the two involved in the contact. Each transmission phase was divided into intervals of 5, 10, and 15 min and each set of intervals was tested.

### Dynamic Analysis

A stochastic agent-based model was run using R (version 3.1.3) (R Core Team, 2015) and the RSiena package (version 1.1-232) (Ripley et al., 2013a) after testing for the different time-scales we had previously defined (i.e., 5, 10, and 15 min). Indeed, changes between two consecutive networks can be too small to rise above the significance threshold, or too large for the model to consider the networks as consecutive stages of the same process. In our case, this lead to an impossibility for the models to converge on our data based on the 5 (few individuals are connected in the matrix) and 15-min (all individuals are connected) intervals. All modeling was thus performed on the 10-min intervals, for which convergence was always successful and satisfying; all *t*-statistics for convergence were inferior to 0.1, suggesting a satisfying estimation of the model (Ripley et al., 2013b).

We checked for the amount of changes between consecutive networks using the Jaccard index, which expresses the similarity between two sets of matrices ranging from 0 (completely different) to 1 (exactly the same). A Jaccard index higher than 0.2 indicates that consecutive networks are similar enough to be considered as successive states of the same network, thus allowing for an RSiena modeling approach (Ripley et al., 2013b). Before running the analysis, we also removed the first time interval from the data; live observations of the flies after they were introduced into the arena showed enhanced activity in all individuals during the first interval of the transmission phase.

The dynamic analysis for weighted networks is not yet implemented in RSiena; we thus performed all the following analysis using binary matrices (Ripley et al., 2013b). The network measures discussed in this study are thus referring to unweighted degrees. We consider these measures just as relevant as their weighted equivalents in our case (see **Table 1**). Indeed, a binary matrix based on degree instead of strength, actually informs on the total number of different individuals that contacted or have been contacted by each focal fly.

**Table 1:** Sum of interactions experienced by 12 female flies (eight informed and four uninformed) during 48 video recorded transmission phases.

	Video ID	Total interaction	Total binarized interaction
**Follow**			
1	Video 7	6897	2478
2	Video 9	3434	1629
3	Video 10	3952	1703
4	Video 11	3524	1485
5	Video 13	2931	1368
6	Video 14	3564	1626
7	Video 23	3877	1622
8	Video 24	3609	1559
9	Video 28	4036	1767
10	Video 30	4287	1803
11	Video 31	5130	2017
12	Video 59	4255	1744
13	Video 65	6098	2207
14	Video 66	6411	2307
15	Video 67	5329	1862
16	Video 68	4314	1897
17	Video 69	3512	1573
18	Video 71	2721	1332
19	Video 73	4689	1813
20	Video 76	4393	1715
21	Video 77	5655	2215
22	Video 78	5468	2151
23	Video 79	6592	2371
24	Video 84	6753	2284
25	Video 89	4600	1903
26	Video 90	6626	2345
27	Video 92	2636	1260
28	Video 97	2072	1121
29	Video 103	5018	1885
**Avoid**			
1	Video 6	5109	1912
2	Video 15	3492	1604
3	Video 21	3345	1545
4	Video 22	4480	1695
5	Video 25	4079	1593
6	Video 26	3952	1749
7	Video 64	6049	2255
8	Video 70	3267	1497
9	Video 72	3041	1463
10	Video 74	4126	1620
11	Video 80	4171	1726
12	Video 83	6146	2252
13	Video 87	4899	1971
14	Video 91	1757	976
15	Video 95	3042	1432
16	Video 96	4107	1694
17	Video 99	5222	1991
18	Video 100	4264	1774
19	Video 103	6026	2114

*Both weighted and binarized matrices for follow (n = 29) and avoid (n = 19) conditions are presented here. Video ID column, indicating the time order at which experiment were run, is also presented*.

### Actor-Oriented Model

The actor-oriented dimension of the model allows us to test hypotheses regarding how individuals affect the network structure by changing their outgoing ties, i.e., who they interact with. However, our study focuses more specifically on two classes of individuals, informed and uninformed flies, and how these classes interact by comparing behaviors at the class level. Moreover, one of the assumptions of the model is that network ties can be regarded as states, rather than events. Conceptually, it is more intuitive to consider a network of flies interacting as a succession of events, one event being described as one interaction between two flies. Another major assumption of RSiena is that the network’s probabilities of change follow a Markov process, i.e., that the current state of a network is the only probabilistic determinant of its dynamic. However, this does not necessarily imply that past states are irrelevant; they can intervene through the influence they have on the current state itself. In our case, this will be reflected by the fact that past interactions will affect an individual’s current behavior, as it is likely to have changed its state along the way (i.e., acquired information) or have a different knowledge of its social environment as it contacts other informed and uninformed flies.

Several methods of estimation have been implemented into Siena since its development: the Method of Moments (Snijders, 2001), the Maximum Likelihood method (Snijders et al., 2010a) and the Bayesian method (Koskinen and Snijders, 2007). While the two latter sets of methods usually yield smaller standard errors for estimates, their use is strongly encouraged in situations with small network datasets and/or very complex models. Considering the size of our data as well as the relative simplicity of our models, the Method of Moments was deemed sufficient and allowed for faster computing calculations (Ripley et al., 2013b). The principle of this procedure is to condition on the first observation; the first observed network (i.e., the network built from observations in the first time interval) is used as the starting point of the simulation, rather than estimated, and thus used to estimate changes between the first interval and successive ones. At each time step, the same procedure is applied until the final interval is reached. We applied the *evaluation function* to determine the probability of change for actors in the network based on the state of the network and on actor behavioral covariates. This function is described as the primary determinant of the probabilities of change of a network, and it can be expressed using the wide range of covariate effects defined in RSiena (Ripley et al., 2013b).

**FIGURE 1:**
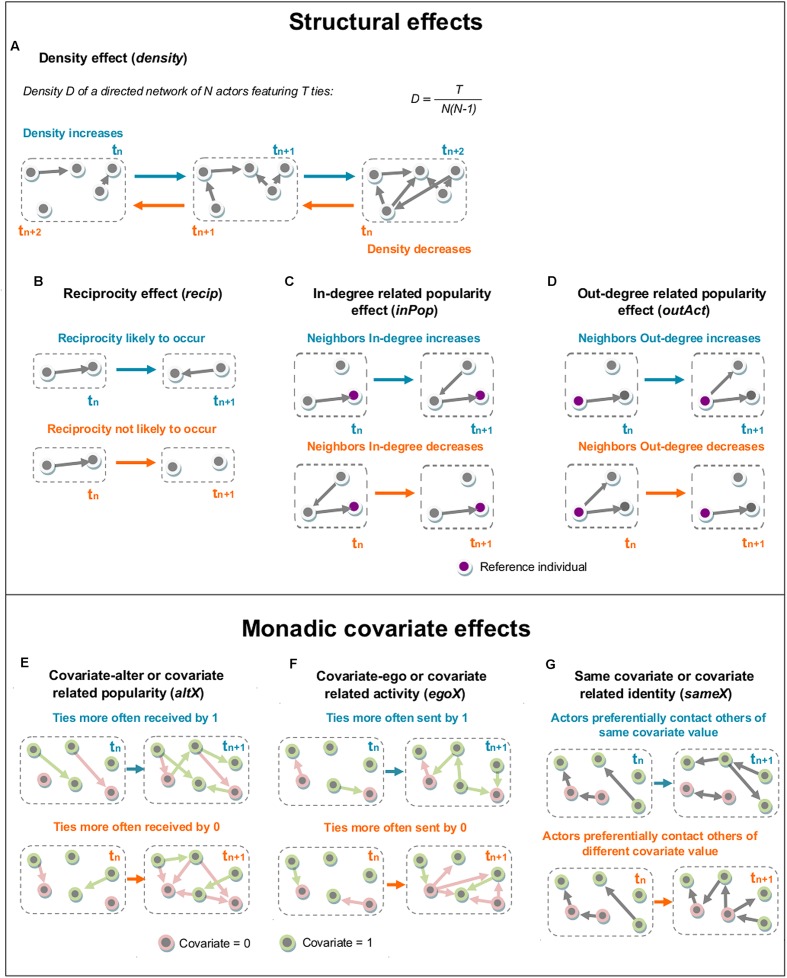
**Interpretation of the RSiena structural effects tested on the “Followed” and “Avoided” data.** Each graph delimited within a single dashed gray box represents an observation of a directed network during a given time interval (denoted by t_n_). Successive states of the network and the dynamics of each effect through time are shown by successive dashed gray boxes (denoted by t_n+1_ and t_n+2_). (1) Structural effects are effects related to network measures only, while monadic covariate effects are related to individual characteristics defined by a binary covariate (here, informed vs. uninformed). Color keys are the same over all figures, with blue elements describing cases where the effect in question has positive and significant dynamics, and orange elements where these are negative and significant. Pink nodes represent uninformed flies (covariate = 0), and green nodes informed ones (covariate = 1). Structural effects are only related to the network: **(A)** The density effect *(density)*, defined by the outdegree of the actors. When significant, it expresses whether density in the network is increasing or decreasing over time, i.e., whether relations are more often created or dissolved. A positive significant statistic (blue) indicates that density overall increases, and a negative significant statistic (orange) that density overall decreases. **(B)** The reciprocity effect *(recip)*, defined by the number of reciprocated interactions, i.e., the number of instances in which the actor of interest also received an interaction from the actor it contacted. When positive (blue), it expresses that an actor is more likely to send an interaction to actors that have previously sent it one and when negative (orange) it represents avoidance. Non-significant values for this effect represent cases in which the reciprocal behavior is random. **(C)** The indegree related popularity effect (*inPop*) reflects the tendency of the neighbors of each actor to receive interactions by others in the network. When significant it underlines the role of neighbors as bridges of information. **(D)** The outdegree related activity effect (*outAct*) reflects the probability of the actor to be contacted by neighbors with a large number of contacts sent. Significant statistics for this measure mean that an individual is largely contacted by highly active individuals. (2) Monadic covariate effects are related to an individual covariate, in our case the class of the actor of interest (informed or uninformed): **(E)** The covariate-alter or covariate related popularity (*altX*), defined by the sum of the covariates over all actors with whom the actor of interest has an interaction. When significant, it expresses which class of actors receives interactions from others more rapidly. For a significant statistic, the interpretation will be that informed flies are contacted by others more rapidly than uninformed flies if it is positive (blue), and *vice versa* if the statistic is negative (orange). **(F)** The covariate-ego or covariate related activity (*egoX*), defined by the actor’s outdegree weighted by its covariate value. When significant, it expresses which class of actors starts interactions more rapidly. For a significant statistic, the interpretation will be that informed flies contact others more rapidly than uninformed ones if it is positive (blue), and *vice versa* if the statistic is negative (orange). **(G)** The same covariate or covariate related identity (*sameX*), defined by the number of interactions of the actor of interest to all other actors who have exactly the same value of covariate (i.e., informed-informed or uninformed-uninformed). When significant, it expresses how likely the actor of interest is to interact with others who share the same covariate value. A positive statistic (blue) will thus express homophily (i.e., actors interact more often with others who have the same covariate value) and a negative one (orange) heterophily (i.e., actors interact more often with others who have a covariate value different from their own).

RSiena allows for the combined analysis of several independent networks and estimation based on repeated measures. Networks are considered independent when they are composed of different sets of actors and when it can be considered that these networks do not influence each other. Such was the case in our study, where new individuals were used for each experiment. Several methods are proposed to achieve this type of analysis. We selected the multi-group analysis for its fast computing time and its estimation of rate parameters for each independent network, as opposed to other methods which yield a single rate parameter for all networks for each interval (Ripley et al., 2013b). These rate parameters express the rate of change between two successive networks, i.e., – the speed at which new interactions between individuals who were not previously interacting occur and existing interactions disappear. In such an actor-based model, several effects can be analyzed: (1) structural effects, describing the variation of the whole structure of the network over time and only depending on the network itself, (2) monadic covariate effects, which use individual characteristics as statuses of individuals in the network, and (3) dyadic covariate effects, typically used to analyzed the effect of more than one actor on the individual network measures (see Ripley et al., 2013b for a detailed description of all the available effects in RSiena). However, because the model implemented by RSiena was constructed with studies of human networks in mind, not all effects are relevant for our purpose. We consequently identified and tested the effects most relevant to our question (**Figure 1**). Each effect was tested using a Wald *t*-test. We followed a two-step procedure; we first tested some pertinent effects in a preliminary global model including both structural (i.e., density, reciprocity, square of contacts sent, and sum of contacts received by neighbors) and monadic (actors hereafter called *ego*, receivers hereafter called *alter*, and homophily) effects (**Figure 1** provides a detailed description of the tested effects). Secondly, in order to better characterize the impact of individual status on information transmission processes, we implemented monadic effects alone (i.e., *ego*, *alter*, and homophily) on time-based subsets of our data. *Density* cannot really be interpreted by itself, as all other statistics are correlated with it; it is included to control for the density of the network, as advised by the RSiena developers (Ripley et al., 2013b). We modeled subsets of increasing size, starting with the first two interaction matrices (i.e., the second and the third time intervals from our original data). Following subsets were generated by incrementing their length by 10 min, or one time interval, each time. Thus, the dynamics of the *t*-statistics for the *ego* and *alter* effects were estimated using two linear models, with time intervals and experimental condition as predictors in each model. We also tested for the presence of a quadratic relationship of the *ego* and *alter* effects with time, comparing linear and quadratic regressions using the *F*-test. A quadratic relationship can suggest the existence of a possible plateau in the relationship between time and the number of contacts sent or received, above which the transmission process stabilizes. We applied a forward stepwise procedure to select our models. To implement the selection we first created a model for each effect previously described and we then aggregated the estimates and we excluded all the non-significant effects. All the models were tested for their goodness of fit to ensure their likelihood in explaining original data by using the “sienaGOF” function from the RSiena package^1^.

## Results

**FIGURE 2:**
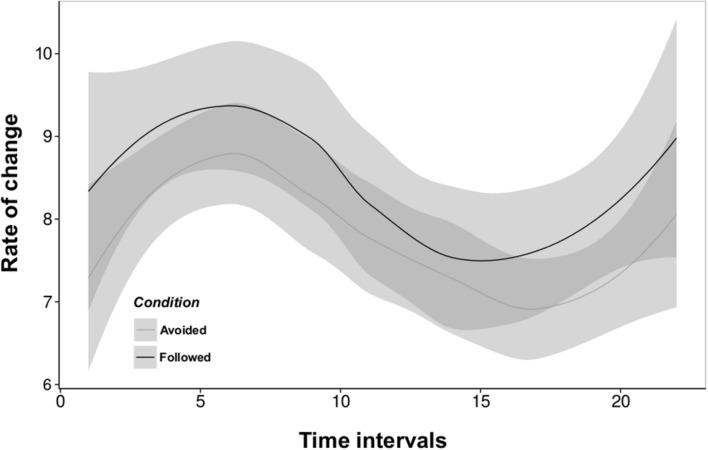
**Rate parameters of the networks estimated from the multi-group analysis in RSiena for each of our two experimental conditions (“Followed” and “Avoided”).** Rate parameters express the number of opportunities for change between successive networks for one given actor. A change is understood as the creation or the deletion of relations among actors during two successive networks. The number of observed changes is, however, always lower than what rate parameters imply; an opportunity for change can be resolved by a ‘no change’ decision, and successive changes can cancel each other out (e.g., create then dissolve a given relation during the same interval). They do not differ between conditions; in both cases actors always have opportunities for change from one network to the next, and although the number of opportunities varies over time, it evolves similarly whether information was followed or not. The success of information transmission is thus not primarily dependent on the opportunities actors get to change their connections to other actors. Best fitted lines for a non-linear model are represented for “Followed” (black) and “Avoided” (grey) conditions. Shaded areas represent the standard errors of the models.

Jaccard indexes were superior to 0.2 in 675 out of 696 and 437 out of 456 10-min matrices used from our “Followed” and “Avoided” conditions respectively, ensuring a sufficient change among consecutive networks to apply our subsequent RSiena analysis. Indeed, mean rate parameters evolve over time as a sinusoidal distribution for both the “Followed” and “Avoided” conditions, meaning that the dynamics of the networks reveal similar, comparable patterns between conditions (**Figure 2**). Applications of the multi-group stochastic estimation procedure performed by RSiena on our experimental data (conditions “Followed” and “Avoided”) yielded models illustrating the dynamics of network measures and the influence of oviposition experience on behavior and network structure. The stepwise model selection procedure yielded a parsimonious model defined by the *density*, *reciprocity*, *alter*, and *ego* effects. For the “Followed” condition model, the square of the number of contacts sent was also retained. Whether information was followed or not, uninformed individuals received interactions from the opposite fly type (*alter* effect) more frequently than informed ones (Followed: *t* = -3.973, *P* < 0.001; Avoided: *t* = -4.103, *P* < 0.001) and they initiated interactions toward the opposite fly type (*ego* effect) significantly more than informed individuals (Followed: *t* = -10.036, *P* < 0.001; Avoided: *t* = -13.449, *P* < 0.001).

Reciprocity, the tendency of individuals to form mutual connections between each other, was always significant both in the “Followed” and “Avoided” conditions, but showed opposite trends: uninformed flies followed the information carried by informed individuals when reciprocity was significantly lower than random (*t* = -12.166, *P* < 0.001), while they avoided it when it was higher (*t* = 10.396, *P* < 0.001). The number of contacts received by neighbors did not influence the transmission process, neither in the “Followed” nor in the “Avoided” conditions (Followed: *t* = -0.493, *P* = 0.622; Avoided: *t* = -0.551, *P* = 0.582).

The more the square of the number of contacts sent (*outdegree activity*) increased, the less likely information was to be followed (*t* = -2.185, *P* = 0.029), meaning that an elevated mobility of flies inside the arena was somehow impeding the acceptance of the information by uninformed individuals. Finally, homophily within classes of flies had no effect on the transmission process, neither in the “Followed” nor in the “Avoided” conditions (Followed: *t* = -0.313, *P* = 0.751; Avoided: *t* = -0.413, *P* = 0.682).

**FIGURE 3:**
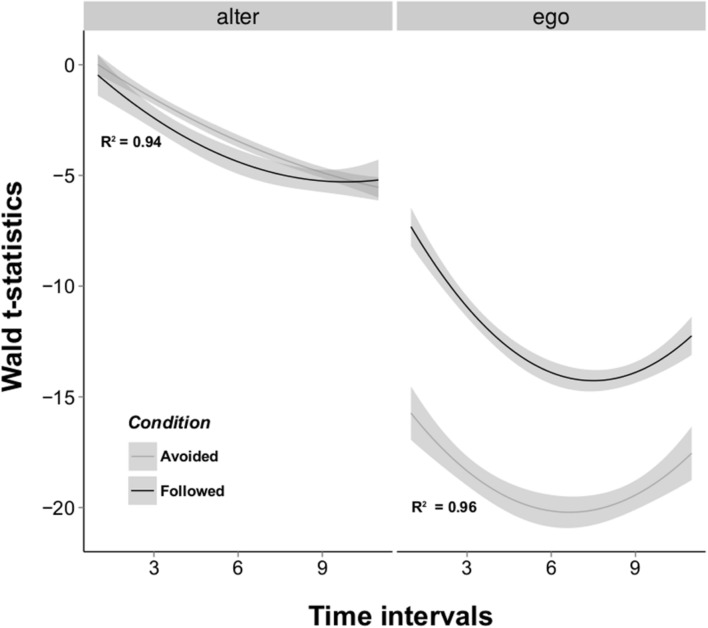
**Wald *t*-test values for the *alter* and *ego* effects obtained by the RSiena model estimation.** The *alter* effect represents which class of actors increases its indegree more rapidly, while the *ego* effect expresses which class increases its outdegree more rapidly. These *t*-statistics were estimated over time intervals of increasing length for the two transmission conditions. Results show that uninformed individuals display both a higher outdegree and indegree than informed individuals (negative *t*-statistics) and that the difference in outdegree between the two classes is more important than in indegree. Best fitted lines for the quadratic models are represented for “Followed” (black) and “Avoided” (grey) conditions. Shaded areas represent the standard errors of the models.

To better evaluate the influence of the *ego* and *alter* effects over time we repeated the RSiena procedure over intervals of increasing lengths, starting from the first 10-min interval and adding successive intervals one by one. An analysis of variance showed that both *ego* and *alter t*-statistics were better explained as a quadratic function of time (ego_quadratic_linear_: *F* = 63.732, *P* < 0.001; alter_quadratic_linear_: *F* = 17.016, *P* < 0.001), meaning that informed and uninformed flies first increase their differences in terms of numbers of contacts started and received, then reduce these behavioral differences over time (**Figure 3**). The difference in the number of contacts received by informed and uninformed flies was larger when information was followed than when it was not (Condition_Followed_Avoided_: *t* = 3.084, *P* < 0.001). There is also a large discrepancy in the magnitude of the *t*-statistics associated with the *ego* effect: the difference in the number of individuals contacted by informed and uninformed flies is constantly smaller in the “Followed” condition (Condition_Followed_Avoided_: *t* = -19.231, *P* < 0.001). These results suggest that a large heterogeneity in the number of contacts sent and received by both fly types drove uninformed flies to choose the opposite oviposition site informed flies were previously trained to choose.

## Discussion

Using data collected on fruit flies in the context of social learning, we have investigated how the behaviors of informed and uninformed individuals could explain the varying success of information transmission, as reflected in the different strategies adopted by uninformed flies after they had interacted with informed individuals.

The RSiena multi-group analysis shows that uninformed flies always contacted and were contacted by more individuals than informed ones. This result is in accordance with a previous work we have done on the same set of flies where we showed an increase in the mean activity level for uninformed flies during transmission phase, probably due to an increased interest in interacting brought upon by flies bringing some novel odors into the uninformed flies’ environment (Battesti et al., 2015). Indeed, uninformed flies were always more active both as sender and receiver. Interestingly, uninformed flies tended to follow the information provided by informed ones when the difference in contacts sent between the two fly types was moderate. This phenomenon occurs either because uninformed flies have contacted fewer individuals, or because informed flies have contacted more. This result suggests that information transmission may occur following an active rather than a passive process, which can be explained both by a search of the information performed by uninformed individuals and/or by an active exchange performed by informed individuals. The active participation of informed flies is not obviously expected following Battesti et al.’s (2015) results. In Battesti et al. (2015), we have shown that the performance of informed flies after the transmission phase was negatively affected by the rate of previous interactions with uninformed individuals, revealing a possible associated cost to information transfer for informed flies. In that study we suggested that the observed transmission process from informed to uninformed flies, and the resulting change in the behavior of informed flies, could be adaptive as the survival rate of larvae might depend on a balance between optimal resource exploitation rate and larval competition. Indeed, an increased number of larvae will exploit the resource more efficiently than a small aggregate, and will be more likely to prevent the development of bacterial and fungal competitors (Rohlfs and Hoffmeister, 2003; Rohlfs, 2005). However, an extremely large number of larvae foraging on the same medium will also impact the per-capita resources available for individual use, thus increasing competition among individuals (Pulliam and Caraco, 1984).

Interesting results were found in relation to the reciprocal behavior of flies: uninformed flies tended to avoid the information brought by informed ones when a large number of reciprocated interactions occur. For information to be transmitted, non-reciprocal contacts are crucial. Network reciprocity has been suggested to negatively affect the formation of smoothed boundaries in clustered population. In particular, in a recent prisoner’s dilemma game developed to study the impact of network reciprocity and individual conformism on cooperation, Szolnoki and Perc (2015) have demonstrated that, starting from a clustered population made of two type of individuals (cooperators and defectors) experiencing a relatively high value of network reciprocity, an increase in the fraction of conformist individuals in the population led to an increase in cooperative behaviors among individuals by smoothing the interaction boundaries among clusters. In accordance to this theoretical work our results suggest that network reciprocity may direct uninformed flies toward an “anti-conformist” site choice underlining the regulatory role, especially as social obstacle, played by high level of reciprocal interactions. It is interesting to note that in a previous work describing the genetic determinants of social structure in different *Drosophila* strains, Schneider et al. (2012) found that olfactory mutant flies (individuals with a severe loss of smell) showed a higher percentage of reciprocated interactions and a disrupted social interaction network compared to wild-type flies. These results may bring additional improvements to the understanding of both the ultimate and proximate factors influencing the efficiency of information transmission processes in this species.

The dynamic network analysis performed over intervals of increasing lengths shows that differences in the estimation of the number of contacts sent and received by informed and uninformed flies best fit a quadratic distribution, with a positive concave curve (**Figure 3**). This suggests that the interaction network stabilized with time through a reduction of the differences between the behaviors of informed and uninformed flies. A large variability in the number of contacts can be caused by the abnormal activity of a few individuals in a network (also known as the friendship paradox; Feld, 1991) which may experience higher rate of interactions in their social milieu. This time-leveling phenomenon is in accordance with the synchronization of activities in fruit flies which has been shown to be affected by social interaction between individuals (Lone and Sharma, 2011).

To the best of our knowledge, this is the first time that an actor-oriented model approach was used to evaluate the correlation between network dynamics and information transmission in animals, suggesting that RSiena might provide useful analytical tools to answer other ecological and evolutionary questions. RSiena allowed us to analyze the dynamics of interaction networks during social transmission experiments and to identify the flies’ involvement in the process of information transfer. The implementation of additional effects taking into account social processes observable in more complex animal societies would make this tool even more useful to biologists studying other species by allowing them to construct complex models to explain the dynamics of their observed interaction networks. For instance, RSiena’s actor-oriented models might be used to estimate the dynamic formation of triadic subgroups (i.e., *transTrip* effect in the RSiena manual) in species experiencing triadic coalitions among group members (e.g., primates Kappeler, 2012; *Corvus corax* Loretto et al., 2012). Moreover, estimation in RSiena is based on the analysis of unweighted networks, meaning that the data used as input for this program reflects the interactions between individuals in the group, but not their frequency. Being able to work directly on weighted networks would allow for the integration of the number of interactions occurring between a given pair of individuals. Because biological processes involving information transmission are likely to rely heavily on repeated interactions between animals (Wei et al., 2015), this improvement would certainly provide even more insight into the mechanisms regulating such processes.

Results obtained in this work show a strong effect of network properties on the future oviposition site choice of uninformed flies. In this context, our results may grant more interest as well because they were obtained from an oversimplification of the repeated interactions occurring between flies in the arena. However, it is possible that in our experiments, uninformed flies may have switched from an uninformed state to an informed state before the transmission was over. Flies may need to pass a threshold of a minimum number of interactions before they can make this switch. Understanding the timing of this switch, and its integration with social interactions, is critical for information transmission studies where a threshold process may occur (Watts, 2002).

The information transmission process is likely to be affected by the proportion of informed and uninformed flies interacting. Previously unpublished experiments run by Mery’ lab (personal communication) have revealed that twice as many informed as uninformed individuals are needed in the arena for the information to be transmitted. It would be interesting in the future to analyze variable proportions of informed and uninformed flies to better evaluate the existence of such a threshold mechanism, which has already been well described theoretically in social learning literature (i.e., see social learning benefit when copying is rare: Boyd and Richerson, 1985; Giraldeau et al., 2002).

Finally, a well-determined subset of videos (i.e., 77% of the total videos run) was used to understand the dynamic effect of network measures on oviposition site choice in this work (i.e., where the proportions of eggs laid by uninformed flies was outside of the [0.2; 0.8] interval). RSiena’s multi-group analysis allows for a parallel comparison of multiple binary networks that can be merged based on clear definitions (follow and avoid information in our case). More studies are needed to understand which network properties affect the remaining random choice we obtained in 23% of the data. In particular, the random outcome obtained in such videos might be caused by different spreading dynamics that could have been actually produced by repeated interactions among individuals. In this context, Relational Event Models (REMs: Tranmer et al., 2015) might be an interesting tool to estimate the impact of multiple repeated interactions on the transmission process. REMs indeed evaluate the sequence of events occurring in each network, allowing also for weighted network analysis and thus possibly explaining the effect of multiple interactions among individuals on future oviposition site choice.

Using *Drosophila* as a model allowed us to make use of the powerful multi-group analysis developed in RSiena while using a substantial data set, obtained from independent repeated experiments. Likewise, studies using different experimental conditions, different mutant strains, or groups with different ecological or physiological characteristics could benefit from a similar approach. However, many studies of animal networks focus on species and social processes for which fewer observations are available, meaning that a multilevel network analysis (such as the multi-group analysis we used here) may not always be possible. However, past uses of RSiena have yielded interesting and valid results, even when repeated experiment cannot be performed (Ullrich et al., 2010; van Zalk et al., 2011). It thus seems that this tool could be used to study a wide range of animal species, varying in group size, social complexity, and access by observers, as recently shown in Ilany et al. (2015). We confirm here that a network dynamic approach is a strong tool for understanding information transmission in a mixed group of flies. This transmission process notably involves specific social behaviors from both informed and uninformed individuals, such as reciprocity between individuals and number of contacts sent or received upon which the success of information diffusion is conditioned.

## Author Contributions

CP and EK carried out the data analysis and paper writing; MB and FM supplied fly videos; JP performed the calculation on the Cloud Computing platform at the IPHC-CNRS in Strasbourg. CS and FM supervised the study. CP, EK, JP, MB, FM, and CS wrote the manuscript. All authors gave final approval for publication.

## Conflict of Interest Statement

The authors declare that the research was conducted in the absence of any commercial or financial relationships that could be construed as a potential conflict of interest.

## Footnotes

1 http://www.inside-r.org/packages/cran/RSiena/docs/plot.sienaGOF
